# Association of Serum Homocysteine with Cardiovascular and All-Cause Mortality in Adults with Diabetes: A Prospective Cohort Study

**DOI:** 10.1155/2022/2156483

**Published:** 2022-10-11

**Authors:** Jingtong Lu, Kegong Chen, Wei Chen, Chang Liu, XingPei Jiang, Zili Ma, Dong Li, Yanjiao Shen, Hai Tian

**Affiliations:** ^1^Department of Cardiovascular Surgery, The Second Affiliated Hospital of Harbin Medical University, Harbin, Heilongjiang, China; ^2^Future Medical laboratory, The Second Affiliated Hospital of Harbin Medical University, Harbin, Heilongjiang, China; ^3^Department of Thoracic Surgery, The Third Hospital of Xiamen, Xiamen, China; ^4^Department of Thoracic Surgery, The First Affiliated Hospital of Anhui Medical University, Hefei, China; ^5^Chinese Evidence-Based Medicine Center, West China Hospital, Sichuan University, Chengdu, Sichuan, China; ^6^Medical Device Regulatory Research and Evaluation Centre, West China Hospital, Sichuan University, Chengdu, Sichuan, China

## Abstract

**Background:**

Homocysteine (Hcy) was implicated in oxidative stress and diabetes biologically. However, the clinical evidence on the link between Hcy level and diabetes is limited and controversial. This study is aimed at investigating the association of serum Hcy with all-cause and cardiovascular mortality in diabetic patients.

**Methods:**

Serum Hcy was measured among 2,286 adults with type 2 diabetes in NHANES 1999-2006. Cox proportional hazard regression was used to estimate hazard ratios (HR) and 95% CIs for the association of Hcy with all-cause and cause-specific mortality.

**Results:**

Over a median follow-up of 11.0 (interquartile range, 8.9-13.4) years, 952 of the 2286 patients with diabetes died, covering 269 (28.3%) cardiovascular deaths and 144 (15.2%) cancer deaths. Restricted cubic spline showed the linear relationship between Hcy and all-cause mortality risk. After multivariate adjustment, higher serum Hcy levels were independently associated with increased risk of all-cause and cardiovascular mortality. Compared with participants in the bottom tertile of Hcy, the multivariate-adjusted HRs and 95% CI for participants in the top quartile were 2.33 (1.64-3.30) for all-cause mortality (*p*_trend_ < 0.001), 2.24 (1.22-4.10) for CVD mortality (*p*_trend_ = 0.017), and 2.05 (0.90-4.69) for cancer mortality (*p*_trend_ = 0.096). The association with total mortality was especially stronger among patients with albuminuria. Serum Hcy significantly improved reclassification for 10-year mortality in diabetic patients (net reclassification index = 0.253 and integrated discrimination improvement = 0.011).

**Conclusions:**

Serum Hcy was associated with risks of all-cause and cardiovascular mortality in diabetic adults. Our results suggested that Hcy was a promising biomarker in risk stratification among diabetic patients.

## 1. Introduction

The incidence of diabetes mellitus and diabetic complications was rapidly increasing, affecting more than 400 million people worldwide [1, 2]. Cardiovascular disease (CVD) remains as the major cause of morbidity and mortality in diabetic patients [3]. The American Diabetes Association (ADA) recently underlines the necessity to improve risk stratification and enable individualized treatment to early prevent morbidity and mortality [4]. Plasma haemoglobin A1c (HbA1c) is of limited prognostic value since it only reflects glucose level over the preceding three months, which cannot develop an effective association with the long-lasting accumulated effects of disease progression [5]. Novel prognostic biomarkers remained required in the management of diabetic patients [6].

Homocysteine (Hcy) is a sulfur-containing amino acid, an intermediate product generated from the metabolism of methionine which is metabolized either by the remethylation process or by the transsulfuration pathway [7, 8]. Numerous studies demonstrated that an increase in serum Hcy was a strong risk factor for cardiovascular diseases [9]. According to a recent meta-analysis, the risk of CVD or stroke was elevated by 10% and 20%, respectively, for each 25% increase in plasma Hcy [10]. Similarly, with a 5 *μ*mol/L increase in Hcy levels, the risk of incident heart disease increased by 52% and mortality increased by 32% [11]. Biologically, Hcy may drive the process of CVD through various mechanisms including blood coagulant properties and oxidative stress-inducing injuries of vascular endothelium and arterial walls [12, 13]. However, most of the evidence was concluded in the nondiabetic setting.

Serum Hcy may be a promising biomarker of vascular complications in diabetes [14]. Several clinical studies indicated that Hcy level was associated with the risk of atherosclerosis and CVD in patients with type2 diabetes mellitus [12, 14]. However, some studies did not note a robust association between Hcy level and diabetes or diabetic complications [15, 16]. In particular, whether serum Hcy predicts mortality risk in diabetic patients was unclear. Given this context, this study is aimed at investigating the relationship between serum Hcy and the risk of all-cause and cause-specific mortality and evaluated the additional predictive value of Hcy for risk stratification of long-term mortality among patients with type 2 diabetes based on a nationally representative sample [17].

## 2. Research Design and Methods

### 2.1. Study Population

**Figure 1:**
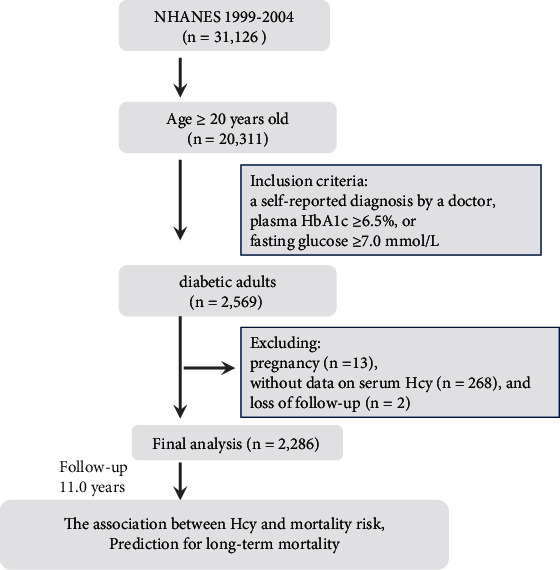
Flow of the study.

Participants in this study were included in the National Health and Nutrition Examination Surveys (NHANES), a stratified and multistage probability sampling study, as described in others and our previous studies [18–20]. NHANES was a nationally representative study of the civilian noninstitutionalized US population of all ages to assess the health and nutritional condition. The protocols and methods of sampling weight and data collection have been published elsewhere [21]. Serum Hcy was determined in four study cycles of NHANES (1999-2000, 2001-2002, 2003-2004, and 2005-2006). Diabetes was defined as a self-reported diagnosis by a doctor, plasma HbA1c ≥ 6.5%, or fasting glucose ≥ 7.0 mmol/L. Among 20,311 adults aged ≥20 years, 2,569 individuals were diagnosed with diabetes. We excluded diabetic adults with pregnancy (*n* = 13), without data on serum Hcy (*n* = 268), and loss of follow-up (*n* = 2). Finally, 2,286 adults with diabetes were included (Figure 1). This study was approved by the research ethics review board of the Centers for Disease Control and Prevention, and all participants provided written informed consent.

### 2.2. Study Exposure: Measurement of Serum Hcy

Serum homocysteine (Hcy) was measured by the Abbott Homocysteine assay, a fully automated fluorescence polarization immunoassay (FPIA) method. Dithiothreitol (DTT) was used to reduce the disulfide bond to albumin and other molecules to free thiol. S-Adenosyl-homocysteine (SAH) hydrolase catalyzes the conversion of Hcy to SAH in the presence of added adenosine. The specific monoclonal antibody and the fluoresceinated SAH analog tracer constitute the FPIA detection system. Hcy level was calculated by the Abbott Axsym® using a machine-stored calibration curve (*r*^2^ = 0.999). This method is linear for homocysteine in the range of 0.8-50 *μ*mol/L with a total coefficient of variation in the range of 3-6%. Samples with results < 2 *μ*mol/L or >15 *μ*mol/L are reanalyzed for confirmation. Samples with total homocysteine concentrations > 50 *μ*mol/L are diluted 10-fold with PBS or FPIA buffer and reanalyzed.

### 2.3. Definition of Covariates

Age, sex, race/ethnicity, smoking status, self-reported cardiovascular disease, cancer, and diabetes-related features and medications at baseline were collected from household interviews using standardized questionnaires [18]. Self-reported cardiovascular disease consisted of coronary heart disease, myocardial infarction, heart failure, and stroke. Duration of diabetes and diabetic peripheral complications (diabetic ulcer/sore, meroparesthesia, or retinopathy) were extracted from diabetic questionnaires [22]. Prescribed medications were recorded during the preceding 30 days. The examination was performed according to standardized protocols and processes. Body mass index (BMI) was calculated as weight (in kilograms) divided by height (in meters) squared. Hypertension was defined by antihypertensive treatment, the average systolic blood pressure ≥ 140 mmHg or the diastolic blood pressure ≥ 90 mmHg at baseline [22]. Blood and urine samples were collected, processed, and transported to central laboratories following validated procedures. Laboratory data on total cholesterol (TC), high-density lipoprotein cholesterol (HDL-C), HbA1c, creatinine, and vitamin B12 were acquired in all cycles. The estimated glomerular filtration rate (eGFR) was calculated using the Chronic Kidney Disease Epidemiology Collaboration using serum creatinine. Urine albumin and creatinine levels were tested with a fluorescent immunoassay and Jaffe rate reaction, respectively. Urine albumin excretion was calculated as urine albumin divided by urine creatinine which was presented as the urine albumin-creatinine ratio (UACR, mg/g) [22].

### 2.4. Study Outcomes

All-cause and cause-specific mortalities were ascertained by the National Death Index with a unique sequence number in the National Center for Health Statistics of US through December 31, 2015 [19]. The leading cause of death was identified using the International Classification of Diseases 10th Revision (ICD-10), including death due to cardiovascular disease (I00-I09, I11, I13, I20-I51, and C00-C97) and malignant neoplasms (C00-C97). The follow-up time was defined from baseline until death or the end of follow-up.

### 2.5. Statistical Analysis

Clustering, stratification, and sampling weights were used to ensure nationally representative estimates according to the analytical guidelines of the NHANES study unless otherwise noted [23]. Baseline characteristics are expressed as weighted means and percentages. The trend of baseline characteristics across the Hcy quartiles was tested by weighted linear regression or logistics regression. Restricted cubic spline based on age- and gender-adjusted Cox proportional hazard model was used to visualize the linear or nonlinear association between serum Hcy and all-cause mortality. Weighted Kaplan-Meier plots were used to visualize all-cause mortality across Hcy strata. Hazard ratios (HRs) and 95% CI for total or cause-specific mortality were assessed by weighted Cox proportional hazard models. Two adjusted models were applied. Model 1 was adjusted for age and sex. Model 2 was further adjusted for race/ethnicity (white, black, Hispanic-Mexican, or other), smoking status (never, quit and current smoking), BMI, hypertension, cancer, CVD, the ratio of TC/HDL-C, vitamin B12, eGFR, plasma HbA1c, metformin, duration of diabetes, UACR, ACEI/ARBs, and diabetic complications. The proportional hazard assumption by estimation of Schoenfeld's residuals was fulfilled for Cox regression model.

In secondary analyses, the association between baseline Hcy and total mortality was ascertained in subgroups by age (<65 and ≥65 years), sex (female and male), B12 (<400 and ≥400 pmol/L), metformin use (no/yes), UACR (<30 and ≥30 mg/g), eGFR (≥ 60 and <60 mL/min/1.73m^2^), duration of diabetes (<10 and ≥10 years), and HbAc1(< 8% and ≥8%) with the fully adjusted model except for stratification factors. The survey-weighted Wald test was adopted to assess the potential interaction.

Several sensitivity analyses were conducted. First, we excluded patients with probable type 1 diabetes who was aged <30 years when first diagnosed as diabetes. Second, we further examined the associations of Hcy with mortality after excluding adults who are first diagnosed with diabetes.

The prediction value of Hcy for 10-year total and heart-specific mortality was assessed in NHANES 1999-2006 via unweighted Cox regression. The reference model was built with currently traditional risk factors, consisting of age, sex, current smoking, BMI, hypertension, cancer, cardiovascular disease, the ratio of TC/HDL-C, eGFR, UACR, HbAc1, diabetic duration, and diabetic complication. The goodness of fit was determined using the likelihood ratio (LR) test, Akaike information criterion (AIC), and Bayesian information criterion (BIC). Harrell's C-index, net reclassification improvement (NRI), and integrated discrimination improvement (IDI) were adopted to assess the incremental discrimination capacity of Hcy based on the reference model [19]. All tests with 2-sided *p* < 0.05 were considered statistically significant using Stata (version 12).

## 3. Results

### 3.1. Participant Characteristics

We investigated serum Hcy in 2,286 patients with type 2 diabetes mellitus. The mean age was 58.9 years, and 50.0% were male diabetic adults. The weighted mean level of serum Hcy was 9.94 (95% CI, 9.62-10.25) *μ*mol/L and skewed distribution (Supplementary Figure 1). Hcy levels were comparable across study years and gender (Supplementary Figure 2). Baseline characteristics of diabetic patients across the quartiles of Hcy levels (Q1: ≤7.34, Q2: 7.33-9.22, Q3: 9.22-11.73, and Q4: >11.73 *μ*mol/L) are presented in Table 1. The patients in higher quartiles were more often older, non-Hispanic whites, and quit smoking and more likely to suffer from chronic disease, including prior cardiovascular disease, cancer, hypertension, and diabetic complications. They also had a longer duration of diabetes, higher UCAR, and lower eGFR.

### 3.2. Correlation between Hcy and Cardiometabolic Biomarkers

Partial correlation coefficients after adjustment for age, sex, and race were used to assess the correlation between Hcy and cardiometabolic biomarkers as shown in Table 2. Serum Hcy was significantly correlated with serum vitamin B12 (*r* = −0.263, *p* < 0.001), whereas the correlation with cardiometabolic biomarkers, including blood pressure, BMI, waist circumference, triglycerides, total cholesterol, HDL-C, LDL-C, plasma glucose, HbA1c, C-peptide, insulin resistance, and C-reactive protein, was insignificant or weak.

### 3.3. Associations of Serum Hcy with All-Cause and Cause-Specific Mortality

Over a median follow-up of 11.0 (interquartile range, 8.9-13.4) years, 952 of the 2286 patients with diabetes died, covering 269 (28.3%) cardiovascular deaths and 144 (15.2%) cancer deaths. The weighted mortality was expressed as the rate per 1000 person-years of follow-up, with 34.2 (95% CI, 31.5-37.3) for all-cause mortality, 9.7 (95% CI, 8.3-11.4) for cardiovascular, and 5.2 (95% CI, 4.2-6.5) for cancer-related mortality.

**Figure 2:**
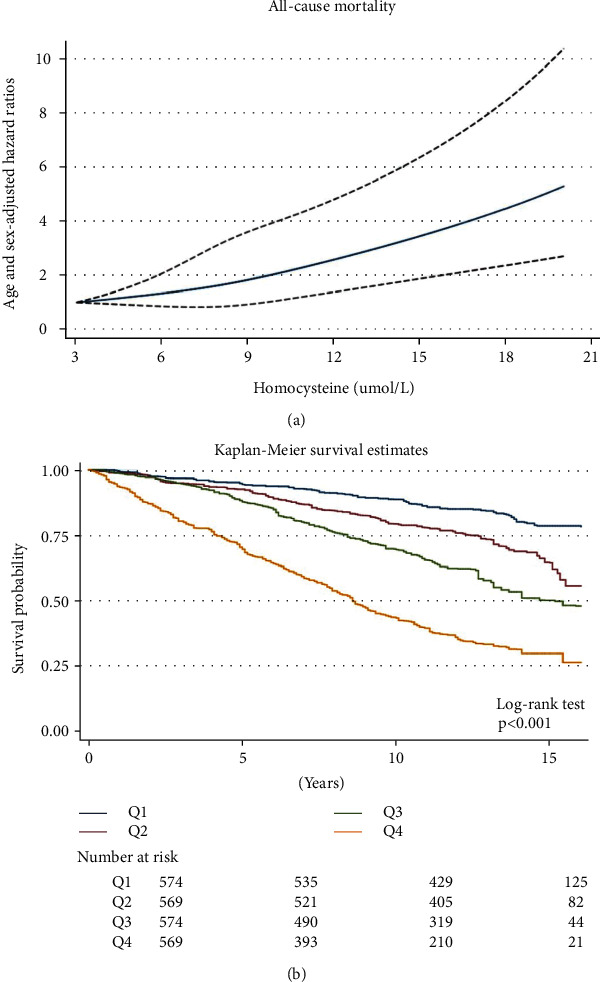
The association of Hcy with total mortality visualized by the restricted cubic spline (a) and Kaplan-Meier curve (b). (a) The restricted cubic spline curve shows the association of Hcy and all-cause mortality. Knots include the 5th, 27.5th, 50th, 72.5th, and 95th percentiles of Hcy. The Cox regression model was adjusted for sex, age, and race/ethnicity. The solid line represents point estimates, and dashed lines represent 95% CIs. The observation number was unweighted.

Restricted cubic spline showed the linear relationship between the doubling of Hcy and all-cause mortality (Figure 2(a)). Consistently, weighted Kaplan-Meier plots also showed significantly elevated risks of all-cause, cardiovascular, and cancer-specific mortality in higher Hcy quartiles (Figure 2(b)). All-cause mortality rates from the lowest to highest quartiles of Hcy were 14.0, 26.3, 39.4, and 81.3 per 1,000 person-years. Compared with the CVD mortality in Q1-Q3 (4.0, 8.2, and 9.8 per 1,000 person-years, respectively), the rate was significantly elevated in Q4 (24.3 per 1,000 person-years). Similar results were noted for cancer-specific mortality (Q1-Q4 2.4, 4.9, 5.5, and 11.0 per 1,000 person-years, respectively).

Weighted multivariable Cox regression analyses were used to assess mortality risk associated with Hcy (Table 3). Serum Hcy at baseline was significantly associated with a higher risk of all-cause, cardiovascular, and cancer-specific mortality, with HR (95% CI) per a doubling of Hcy 2.38 (1.88-3.00, *p* < 0.001), 2.52 (1.91-3.33, *p* < 0.001), and 1.93 (1.50-2.49, *p* < 0.001), respectively. The age- and sex-adjusted HR per doubling Hcy for total and cardiovascular mortality was significant, while for cancer mortality, it was insignificant. After multivariate adjustment for the various confounders, the relationship between log2 transformed Hcy with all-cause and cardiovascular mortality remained significant (each *p* ≤ 0.018), with a 61% and 53% increase in risk per a doubling of Hcy, respectively. Consistently, the mentioned associations across Hcy quartiles remained significant. The adjusted HRs (95% CIs) from the bottom quartile to the top quartile of serum Hcy were 1.00 (reference), 1.13 (0.82-1.57), 1.39 (1.03-1.88), and 2.33 (1.64-3.30) for all-cause mortality (*p* for trend < 0.001) and 1.00 (reference), 1.10 (0.65-1.88), 1.25 (0.74-2.12), and 2.24 (1.22-4.10) for CVD mortality (each *p* trend ≤ 0.017). The increased cardiovascular mortality associated with higher Hcy was mainly attributed to heart disease (Supplementary Table 1). By contrast, the association between mortality due to cancer and Hcy quartiles was insignificant.

### 3.4. Stratification and Additional Analyses

**Figure 3:**
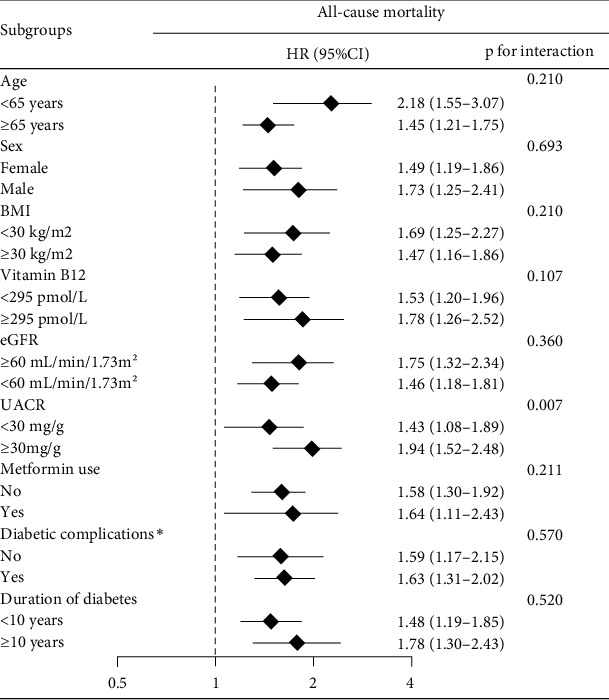
Stratification analysis on HRs of total mortality per doubling of Hcy. HR (95% CI) was estimated with weighted Cox regression adjusted for model 2 except the corresponding subgroup factors. *p* value < 0.05 represents a significance for the interaction of stratification factors for the association of a doubling in Hcy with mortality.

In stratified analyses, the association of serum Hcy at baseline with increased risk of all-cause mortality was largely consistent in most subgroups (Figure 3). A significant interaction in all-cause mortality was noted between serum Hcy and UACR (*p* for interaction = 0.002). The HRs per a doubling of Hcy for all-cause mortality were 1.43 (1.08-1.89) among patients with UACR <30 *μ*g/mg versus 1.94 (1.52-2.48) among those with UACR ≥30 *μ*g/mg. However, there is no significant interaction in mortality between metformin use and serum Hcy.

Excluding individuals with probable type 1 diabetes who were diagnosed as diabetes before 20 years of age and treated with only insulin, a robust relationship between serum Hcy and mortality risk was still identified (Supplementary Table 2). We additionally excluded participants who were first diagnosed with diabetes and noted a significant relationship between serum Hcy and mortality (Supplementary Table 3). For sensitivity analysis, serum Hcy was categorized according to the predefined definition of hyperhomocysteinemia (>15 *μ*mol/L), and the association remained statistically significant (Supplementary Table 4). Compared with diabetic patients with Hcy levels ≤15 *μ*mol/L, the adjusted HRs (95% CIs) for all-cause mortality among those with levels >15 *μ*mol/L were 1.91 (1.47-2.48) and 2.37 (1.50-3.74), respectively (both *p* < 0.001).

### 3.5. Prognostic Value of Serum Hcy

**Figure 4:**
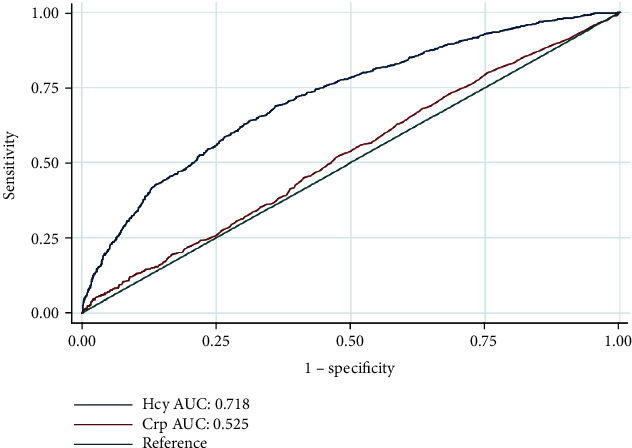
The receiver operating characteristic curves of Hcy and CRP to predict 10-year all-cause mortality risk in diabetic adults.

Using a single biomarker to predict 10-year mortality risk, Hcy had a larger area under the ROC curve than CRP (AUC-ROC 0.718 versus 0.525, Figure 4). Furthermore, the additional value of Hcy for risk prediction of long-term mortality was determined among diabetic patients from NHANES 1999–2006 (Table 4). Adding Hcy to the reference model did not substantially increase Harrell's C-index. A significant improvement in reclassification was noted when adding Hcy to the reference model for all-cause death as measured by NRI and IDI (0.253 and 0.011, respectively; *p* < 0.001 for the likelihood ratio test). However, CRP did not significantly improve predictive performance for mortality based on conventional clinical factors (NRI 0.188 and IDI 0.003, respectively; *p* = 0.074 for likelihood ratio test).

## 4. Discussion

This study investigated the association of serum Hcy with all-cause and cause-specific mortality in adults with type 2 diabetes. Serum Hcy was robustly associated with increased risk of all-cause and heart-related mortality after adjustment for traditional risk factors, whereas the incremental predictive value of Hcy for 10-year mortality risk was significantly on top of the common risk stratification model. To our best knowledge, this study initially demonstrated that Hcy concentration was independently associated with all-cause and cardiovascular mortality in diabetic adults, and Hcy provided an incremental prognostic value to predict long-term mortality beyond the well-established inflammation biomarker C-reactive protein.

The association between Hcy and mortality risk in diabetic patients has not been well established. A large number of previous studies have reported that Hcy was closely related to the development and progression of CVD, cancer, and neurodegenerative diseases [7, 9, 17, 24, 25]. However, most of the investigations concerning the clinical association between Hcy level and mortality risk were mainly reported in a nondiabetic setting [9, 20, 26]. According to the previous two pooled analyses for prospective cohort studies, increased concentrations of serum Hcy were an independent predictor of risks of cardiovascular and all-cause mortality, and the risk was more pronounced in the elderly [11, 27]. Further, the dose-response meta-analysis suggested that increased Hcy was linearly related to the risk of mortality in the general population, with all-cause mortality elevated by 33.6% for each 5 *μ*mol/L increase of serum Hcy [27]. Consistently, among patients with acute coronary syndrome or stroke, elevated Hcy at baseline was also significantly associated with an increased risk of MACE and all-cause mortality [28]. The data on the relationship between Hcy levels and mortality risk in diabetic patients was limited. Looker et al. investigated the relationship between baseline Hcy and mortality during a median follow-up of 15.7 years among 396 diabetic Pima Indians aged 40 years or older [15]. Serum Hcy was positively associated with all-cause and diabetes-/nephropathy-associated mortality in adults with type 2 diabetes. After adjustment for creatinine and other confounders, the association between Hcy and mortality became nonsignificant. However, the population observed in this study was recruited between 1982 and 1985, and epidemiological characteristics of diabetes patients may vary over the past decades [1]. In addition, the study population was limited to Pima Indians which may limit the extrapolation. The higher correlation between Hcy and serum creatinine in their analysis (correlation coefficient = 0.50) than our study (correlation coefficient = 0.35) also supported this point. Ndrepepa et al. observed 507 patients with type 2 diabetes and coronary artery disease between 2000 and 2001 [29]. Although the K-M survival curve showed that accumulative cardiovascular mortality was increased in patients with higher Hcy, the relationship was not significant according to multivariable Cox regression analysis. The limited sample size and cardiovascular deaths (*n* = 62) may limit its statistical efficiency, and the nonlinearity relationship was not considered [29]. In this nationally representative prospective cohort of 2,286 adults with T2DM, we firstly provided evidence that Hcy was independently associated with long-term all-cause and cardiovascular mortality in diabetic patients. Our results supported further evaluation of the clinical prognostic evaluation of Hcy and the potential value for treatment guidance.

The biological role of Hcy in the progression of diabetes and diabetic complications was demonstrated by a large number of studies. Hcy accumulation may be involved in the development of diabetes via aggravating insulin resistance (IR) and macro-/microvascular endothelial dysfunction [30, 31]. The underlying mechanism could be related to the oxidative stress effect of homocysteine in the cardiovascular system [17, 32]. In our findings, the association between serum Hcy and mortality risk was mainly attributed to the increased cardiovascular deaths. That may be explained by previous numerous biological studies that Hcy can lead to the pathogenesis of CVD via multiple mechanisms such as its disadvantageous effects on smooth muscle cells and endothelium with subsequent alterations in arterial function and structure [33, 34]. Oxidative stress as a critical mediator of Hcy increase may promote the proliferation of vascular smooth muscle cells, endothelial dysfunction, platelet activation, and decreased elasticity of the arterial wall [35]. The development and progression of diabetes have been linked with redox disorder, and therefore, serum Hcy seems to be promising biomarker for the risk stratification in diabetic patients [36]. Interestingly, our findings suggested that based on traditional risk factors, Hcy may improve risk stratification of 10-year mortality in diabetic patients beyond inflammatory marker C-reactive protein. The prognostic implication of Hcy in diabetic patients warrants further investigation.

However, whether Hcy is a mediator of cardiovascular pathophysiology remains controversial [10, 33]. Some studies have proved that lowering Hcy cannot yield significant cardiovascular benefits [37]. Part of the explanation may be that the advancement in cardiovascular secondary prevention has masked the benefits of reducing Hcy. Indeed, numerous biological studies demonstrated that Hcy participated in the redox disorder associated with diabetes and diabetic complications and reduced Hcy delayed oxidative stress of multiple organ injuries in mice [36, 38]. Clinical evidence also supported that the level of oxidative stress was significantly elevated in diabetic patients [17]. Further research is needed to prove the direct link between Hcy intervention and diabetes progression. A fundamental part of these studies should identify whether elevated Hcy is the cause or the result of the pathophysiological change and whether it is related to the progression of cardiovascular complications in diabetes. Altogether, taking into account the broad availability and cost-efficacy of Hcy detection, it could be a good screening biomarker for poor prognosis among diabetic patients.

The strengths of our analysis include the use of nationally representative data of US adults with diabetes, a prospective study design, and validated protocols. Several limitations need to be considered. First, causality cannot be concluded due to the observational study design. The genetic variants in Hcy metabolism-related genes may provide more information for the causal relationship between Hcy and outcomes. Second, information on distinguishing type 1 and type 2 diabetes was lacking in this study, whereas oxidative stress is one of the common characteristics in both type 1 and type 2 diabetes. Our findings underline Hcy as a potentially promising biomarker for the progression and poor outcomes of diabetes. Third, although potential confounding factors were adjusted to the greatest extent, we could not completely rule out the possibility of residual confounding unknown. Forth, Single detection of serum Hcy may not accurately assess the long-term levels during the follow-up, but that tends to attenuate results toward the null. Fifth, due to the lower incidence of stroke-related deaths, the association between serum Hcy and stroke-specific mortality should be interpreted cautiously and needs further validation [39].

In conclusion, we found that higher serum Hcy concentrations were significantly associated with increased all-cause and cardiovascular mortality in a nationally representative sample of diabetic adults. Moreover, serum Hcy improves risk stratification in patients with diabetes outmatched C-reactive protein. These findings support further investigation of the potential benefits of lowering Hcy in the prevention of premature death among diabetes patients.

## Figures and Tables

**Table 1:** Baseline characteristics of diabetic patients across quartiles of serum homocysteine.

Variables	Homocysteine (*μ*mol/L)				
	Quartile 1	Quartile 2	Quartile 3	Quartile 4	*p* for trend
	(*n* = 574)	(*n* = 569)	(*n* = 574)	(*n* = 569)	
Homocysteine (*μ*mol/L)	6.14 ± 0.05	8.26 ± 0.03	10.39 ± 0.05	16.67 ± 0.41	
Age (years)	50.21 ± 0.88	58.14 ± 0.76	62.92 ± 0.73	66.72 ± 0.81	<0.001
Male (%)	36.6	51.35	55.54	59.59	<0.001
Race/ethnicity (%)					
Non-Hispanic white	53.04	66.18	66.6	69.64	<0.001
Non-Hispanic black	15.51	13.58	14.23	18.48	0.270
Hispanic-Mexican	13.18	6.842	6.373	4.386	<0.001
Other ethnicity	18.26	13.4	12.8	7.496	<0.001
Smoking status					<0.001
Never smoking	60.07	47.05	42.28	39.35	
Former smoker	22.25	33.85	36.48	43.65	
Current smoker	17.68	19.1	21.23	17	
Physical activity (%)					<0.001
Inactive	43.59	49.98	49.47	65.81	
Moderate activity	30.07	31.61	33.15	27.5	
Vigorous activity	26.34	18.41	17.39	6.69	
BMI (kg/m^2^)	33.20 ± 0.45	32.78 ± 0.42	31.41 ± 0.39	31.41 ± 0.50	0.001
Hypertension (%)	54.24	69.98	72.39	80.75	<0.001
Waist circumference (cm)	108.36 ± 1.05	109.66 ± 0.90	108.33 ± 0.93	109.04 ± 1.09	0.860
Cardiovascular disease (%)	12.65	23.12	31.09	44.03	<0.001
Cancer (%)	7.008	14.68	15.1	17.04	<0.001
TC/HDL-C ratio	2.2 ± 0.10	2.3 ± 0.23	2.28 ± 0.18	2.29 ± 0.17	0.672
HbA1c (%)	7.76 ± 0.13	7.41 ± 0.08	7.29 ± 0.12	6.98 ± 0.10	<0.001
Plasma glucose (mmol/L)	9.10 ± 0.22	8.40 ± 0.18	8.06 ± 0.25	7.97 ± 0.21	<0.001
Insulin (pmol/L)	120.92 ± 7.53	151.48 ± 21.50	131.63 ± 10.63	140.78 ± 10.55	0.340
HbA1c (mmol/mol)	61.36 ± 1.42	57.49 ± 0.88	56.16 ± 1.31	52.79 ± 1.06	<0.001
eGFR (mL/min per 1.73m^2^)	102.88 ± 1.23	89.95 ± 0.99	78.69 ± 1.31	61.02 ± 1.34	<0.001
UACR (mg/g)	72.33 ± 22.72	117.03 ± 23.07	110.76 ± 18.86	310.24 ± 47.51	<0.001
C-reactive protein (mg/dL)	0.78 ± 0.06	0.72 ± 0.07	0.57 ± 0.04	0.70 ± 0.06	0.085
C-peptide (nmol/L)	1.17 ± 0.10	1.25 ± 0.06	1.07 ± 0.06	1.30 ± 0.07	0.653
Metformin use (%)	34.17	31.14	33.4	25.72	0.041
Diabetic complications (%)	31.57	33.75	36.92	47.82	<0.001
Retinopathy (%)	16.99	19.26	17.23	25.32	0.021
Foot ulcer (%)	4.41	2.746	6.855	9.399	0.006
Peripheral neuropathy (%)	19.53	21.6	25.06	31.77	0.001
Duration of diabetes (year)	7.84 ± 0.53	7.83 ± 0.54	9.67 ± 0.79	12.24 ± 0.92	<0.001
Serum B12 (pmol/mL)	529.15 ± 60.63	410.56 ± 10.52	376.62 ± 10.63	432.16 ± 73.35	0.189
Serum folate (nmol/L)	38.11 ± 2.67	38.34 ± 3.58	37.48 ± 1.52	36.91 ± 1.87	0.653

Data are represented as the weighted proportion (%) or mean ± SE. *p* for trend was estimated with linear regression for continuous variables and with logistic regression for categorical variables. BMI: body mass index; CVD: cardiovascular disease; eGFR: estimated glomerular filtration rate; HDL-C: high-density lipoprotein cholesterol; HOMA-IR: homeostasis model assessment for insulin resistance; Hcy: homocysteine; TC: total cholesterol; UACR: urinary albumin-creatinine ratio.

**Table 2:** Partial correlation of serum Hcy with cardiometabolic biomarkers in diabetic adults.

	Partial coefficient	*p* value
Systolic BP (mmHg)	0.030	0.171
Diastolic BP (mmHg)	-0.049	0.022
BMI (kg/m^2^)	0.022	0.304
Waist circumference (cm)	0.035	0.106
Triglycerides∗ (mmol/L)	0.060	0.004
Total cholesterol (mmol/L)	0.018	0.385
HDL-C (mmol/L)	0.043	0.041
LDL-C (mmol/L)	-0.037	0.259
Glucose (mmol/L)	-0.063	0.003
HbA1c (%)	-0.088	<0.001
C-peptide (nmol/L)	0.045	0.204
Insulin (pmol/L)	0.034	0.263
HOMA-IR index	-0.007	0.830
Serum creatinine (*μ*mol/L)	0.348	<0.001
Serum vitamin B12 (pmol/L)	-0.263	<0.001
Serum folate (nmol/L)	-0.045	0.032
C-reactive protein (mg/dL)	0.010	0.641

Partial correlation coefficients were estimated by the Pearson analysis adjusted for age, sex, and race. *p* value ≤ 0.003 was considered significant after Bonferroni's correction. BMI: body mass index; BP: blood pressure; HDL-C: high-density lipoprotein cholesterol; LDL-C: low-density lipoprotein cholesterol.

**Table 3:** HR (95% CI) associated with Hcy for all-cause, cardiovascular, and cancer mortality in adults with diabetes.

All-cause deaths	Doubling in Hcy	*p* value	Q1 (*n* = 574)	Q2 (*n* = 569)	Q3 (*n* = 574)	Q4 (*n* = 569)	*p* for trend
Mortality rate per 1000 person-years	34.2 (31.5-37.3)^∗^		14.0 (11.1-18.0)	26.3 (21.9-31.7)	39.4 (33.8-46)	81.3 (71.2-93)	
Unadjusted model	2.38 (1.88-3.00)^#^	<0.001	1 (ref.)	1.93 (1.42-2.63)	2.98 (2.24-3.97)	6.46 (4.82-8.66)	<0.001
Model 1	1.77 (1.44-2.17)	<0.001	1 (ref.)	1.29 (0.94-1.77)	1.63 (1.28-2.07)	2.90 (2.19-3.84)	<0.001
Model 2	1.61 (1.34-1.92)	<0.001	1 (ref.)	1.13 (0.82-1.57)	1.39 (1.03-1.88)	2.33 (1.64-3.30)	<0.001
*Cardiovascular deaths*							
Mortality rate per 1000 person-years	8.3 (11.4-11.4)		4.0 (2.5-6.6)	8.2 (6.1-11.4)	9.6 (7.1-13.1)	24.3 (19-31.5)	
Unadjusted	2.52 (1.91-3.33)	<0.001	1 (ref.)	2.15 (1.23-3.74)	2.61 (1.44-4.73)	7.00 (4.05-12.12)	<0.001
Model 1	1.82 (1.34-2.48)	<0.001	1 (ref.)	1.30 (0.75-2.26)	1.20 (0.66-2.17)	2.51 (1.34-4.69)	0.007
Model 2	1.53 (1.08-2.16)	0.018	1 (ref.)	1.25 (0.74-2.12)	1.10 (0.65-1.88)	2.24 (1.22-4.10)	0.017
*Cancer-related deaths*							
Mortality rate per 1000 person-years	5.2 (4.2-6.5)		2.4 (1.4-4.5)	4.9 (3.1-8.0)	5.5 (3.6-8.8)	11.0 (7.9-15.7)	
Unadjusted	1.93 (1.50-2.49)	<0.001	1 (ref.)	2.02 (0.92-4.44)	2.27 (1.06-4.85)	4.60 (2.40-8.83)	<0.001
Model 1	1.35 (0.99-1.83)	0.056	1 (ref.)	1.38 (0.6-3.13)	1.26 (0.55-2.88)	2.12 (1.07-4.2)	0.030
Model 2	1.50 (0.94-2.40)	0.087	1 (ref.)	0.96 (0.40-2.35)	0.92 (0.35-2.39)	2.05 (0.90-4.69)	0.096

^∗^Weighted mortality was expressed as a rate per 1000 person-years of follow-up and 95% CI. ^#^HR (95% CI) was estimated by weighted Cox regression analyses. Model 1 was adjusted for age and sex (*n* = 2,286). Model 2 was additionally adjusted for race/ethnicity, smoking, BMI, hypertension, cancer, CVD, TC/HDL-C ratio, lipid-lowering agents, antiplatelet, vitamin B12, eGFR, HbA1c, metformin, duration of diabetes, UACR, ACEI/ARBs, and diabetic complications (*n* = 2,050).

**Table 4:** Predictive value of baseline Hcy for 10-year total and heart-related mortality in patients with preexisting diabetes.

Model	Reference	Reference+Hcy	Reference+CRP
Likelihood ratio test	—	<0.001	0.074
AIC	11118.2	11106.1	11117.0
BIC	11185.9	11179.5	11190.3
Harrell's C-index	0.763	0.766	0.764
NRI	—	0.253	0.188
IDI	—	0.011	0.003

^∗^Reference model included age, sex, smoking status, BMI, hypertension, cancer, cardiovascular disease, the ratio of TC/HDL-C, eGFR, UACR, HbAc1, diabetic duration, and diabetic complications. All statistics were estimated based on the unweighted logistic regression analysis. Each additional model is compared to the reference model. AIC: Akaike information criterion; BIC: Bayesian information criterion; CRP: C-reactive protein; Hcy: homocysteine; IDI: integrated discrimination improvement; NRI: net reclassification index.

## Data Availability

All data used were obtained from the NHANES study (http://www.cdc.gov/nchs/nhanes). Data are available on reasonable request from the website or authors.

## Supplementary Materials

Supplementary Materials Supplementary Table 1: the association of Hcy with mortality due to stroke and cardiac disease in adults with diabetes. Supplementary Table 2: sensitivity analysis for the association between serum Hcy and mortality after excluding probable type 1 diabetes. Supplementary Table 3: sensitivity analysis for the association between serum Hcy and mortality after excluding participants with first-diagnosed diabetes. Supplementary Table 4: the association between serum hyperhomocysteinemia and mortality in diabetic adults.
