# Supplementary material for: Association of Serum Homocysteine with Cardiovascular and All-Cause Mortality in Adults with Diabetes: A Prospective Cohort Study
Source: Oxid Med Cell Longev. 2022 Oct 11;2022:2156483. doi: 10.1155/2022/2156483 (PMC9578792; doi:10.1155/2022/2156483)
Supplement: Supplementary Materials — Supplementary Table 1: the association of Hcy with mortality due to stroke and cardiac disease in adults with diabetes. Supplementary Table 2: sensitivity analysis for the association between serum Hcy and mortality after excluding probable type 1 diabetes. Supplementary Table 3: sensitivity analysis for the association between serum Hcy and mortality after excluding participants with first-diagnosed diabetes. Supplementary Table 4: the association between serum hyperhomocysteinemia and mortality in diabetic adults. [file 2156483.f1.docx]

## Supplementary Table 1.The association of Hcy with mortality due to stroke and cardiac disease in adults with diabetes

| **Mortality** | Doubling in Hcy | p value | Q1 (n=574) | Q2 (n=569) | Q3 (n=574) | Q4 (n=569) | p for trend |
| --- | --- | --- | --- | --- | --- | --- | --- |
| **Cardiac disease** |  |  |  |  |  |  |  |
| Unadjusted | 2.54 (1.90-3.41) | <0.001 | 1(ref.) | 1.68 (0.92-3.07) | 2.13 (1.11-4.06) | 6.12 (3.48-10.76) | <0.001 |
| Model 1 | 1.92 (1.38-2.67) | <0.001 | 1(ref.) | 1.05 (0.58-1.89) | 1.03 (0.53-2.00) | 2.34 (1.22-4.51) | 0.013 |
| Modle 2 | 1.67 (1.14-2.45) | 0.009 | 1(ref.) | 1.01 (0.57-1.78) | 0.84 (0.51-1.72) | 2.08 (1.09-3.95) | 0.032 |
| **Stroke** |  |  |  |  |  |  |  |
| Unadjusted | 2.42 (1.76-3.34) | <0.001 | 1(ref.) | 10.16 (2.46-41.92) | 10.85 (3.31-35.52) | 22.31 (7.54-66) | <0.001 |
| Model 1 | 1.45 (0.86-2.46) | 0.158 | 1(ref.) | 5.41 (1.39-20.99) | 3.86 (1.07-13.96) | 5.82 (1.8-18.83) | 0.204 |
| Modle 2 | 0.88 (0.42-1.91) | 0.759 | 1(ref.) | 4.89 (1.21-19.84) | 3.50 (0.97-12.69) | 5.03 (1.46-17.31) | 0.289 |

HR (95% CI) was estimated by weighted Cox regression analyses. Model 1 was adjusted for age and sex. Model 2 was additionally adjusted for race/ethnicity, smoking, BMI, hypertension, cancer, CVD, TC/HDL-C ratio, lipid-lowering agents, antiplatelet, Vitamin B12, eGFR, HbA1c, metformin, duration of diabetes, UACR, ACEI/ARBs, and diabetic complications.

## Supplementary Table 2. Sensitivity analysis for the association between serum Hcy and mortality after excluding probable Type 1 diabetes

| serum Hcy | HR (95%CI) | p value | HR (95%CI) | p value | HR (95%CI) | p value |
| --- | --- | --- | --- | --- | --- | --- |
| Doubling in Hcy | 2.38 (1.87-3.03) | <0.001 | 1.74 (1.42-2.14) | <0.001 | 1.57 (1.32-1.86) | <0.001 |
| Q1 | 1(ref.) |  | 1(ref.) |  | 1(ref.) |  |
| Q2 | 1.91 (1.40-2.61) | <0.001 | 1.30 (0.95-1.78) | 0.105 | 1.14 (0.82-1.58) | 0.429 |
| Q3 | 2.94 (2.20-3.93) | <0.001 | 1.62 (1.28-2.06) | <0.001 | 1.39 (1.03-1.86) | 0.029 |
| Q4 | 6.43 (4.77-8.66) | <0.001 | 2.84 (2.16-3.75) | <0.001 | 2.26 (1.62-3.15) | <0.001 |
|  |  | <0.001* |  | <0.001* |  | <0.001* |

HR (95% CI) was estimated by weighted Cox regression analyses. Model 1 was adjusted for age and sex. Model 2 was additionally adjusted for race/ethnicity, smoking, BMI, hypertension, cancer, CVD, TC/HDL-C ratio, lipid-lowering agents, antiplatelet, Vitamin B12, eGFR, HbA1c, metformin, duration of diabetes, UACR, ACEI/ARBs, and diabetic complications.

## Supplementary Table 3. Sensitivity analysis for the association between serum Hcy and mortality after excluding participants with first-diagnosed diabetes

| Serum Hcy | HR (95%CI) | P value | HR (95%CI) | P value | HR (95%CI) | P value |
| --- | --- | --- | --- | --- | --- | --- |
| Doubling in Hcy | 2.31 (1.78-3.01) | <0.001 | 1.74 (1.38-2.20) | <0.001 | 1.58 (1.28-1.95) | <0.001 |
| Q1 | 1.00 (ref.) |  | 1.00 (ref.) |  | 1.00 (ref.) |  |
| Q2 | 1.86 (1.32-2.62) | 0.001 | 1.31 (0.93-1.84) | 0.124 | 1.06 (0.73-1.53) | 0.761 |
| Q3 | 3.00 (2.26-3.98) | <0.001 | 1.74 (1.37-2.19) | <0.001 | 1.47 (1.10-1.98) | 0.011 |
| Q4 | 5.94 (4.36-8.08) | <0.001 | 2.85 (2.10-3.87) | <0.001 | 2.23 (1.55-3.20) | <0.001 |

HR (95%CI) was estimated with weighted Cox regression adjusted for age, sex, race/ethnicity, smoking, BMI, hypertension, cancer, CVD, TC/HDL-C ratio, lipid-lowering agents, antiplatelet, Vitamin B12, eGFR, HbA1c, metformin, duration of diabetes, UACR, ACEI/ARBs, diabetic complications and CRP.

## Supplementary Table 4. The association between serum hyperhomocysteinemia and mortality in diabetic adults

|  | All-cause mortality | | Cardiovascular mortality | |
| --- | --- | --- | --- | --- |
|  | HR (95%CI) | p value | HR (95%CI) | p value |
| Crude | 3.78 (2.94-4.86) | <0.001 | 5.50 (3.63-8.34) | <0.001 |
| Model 1 | 2.42 (1.93-3.02) | <0.001 | 3.29 (2.13-5.07) | <0.001 |
| Model 2 | 1.91 (1.47-2.48) | <0.001 | 2.37 (1.50-3.74) | <0.001 |

HR (95% CI) was estimated with weighted Cox regression adjusted for age, sex, race/ethnicity, smoking, BMI, hypertension, cancer, CVD, TC/HDL-C ratio, lipid-lowering agents, antiplatelet, Vitamin B12, eGFR, HbA1c, metformin, duration of diabetes, UACR, ACEI/ARBs, diabetic complications and CRP.

## Supplementary Figure 1. The histogram of serum Hcy


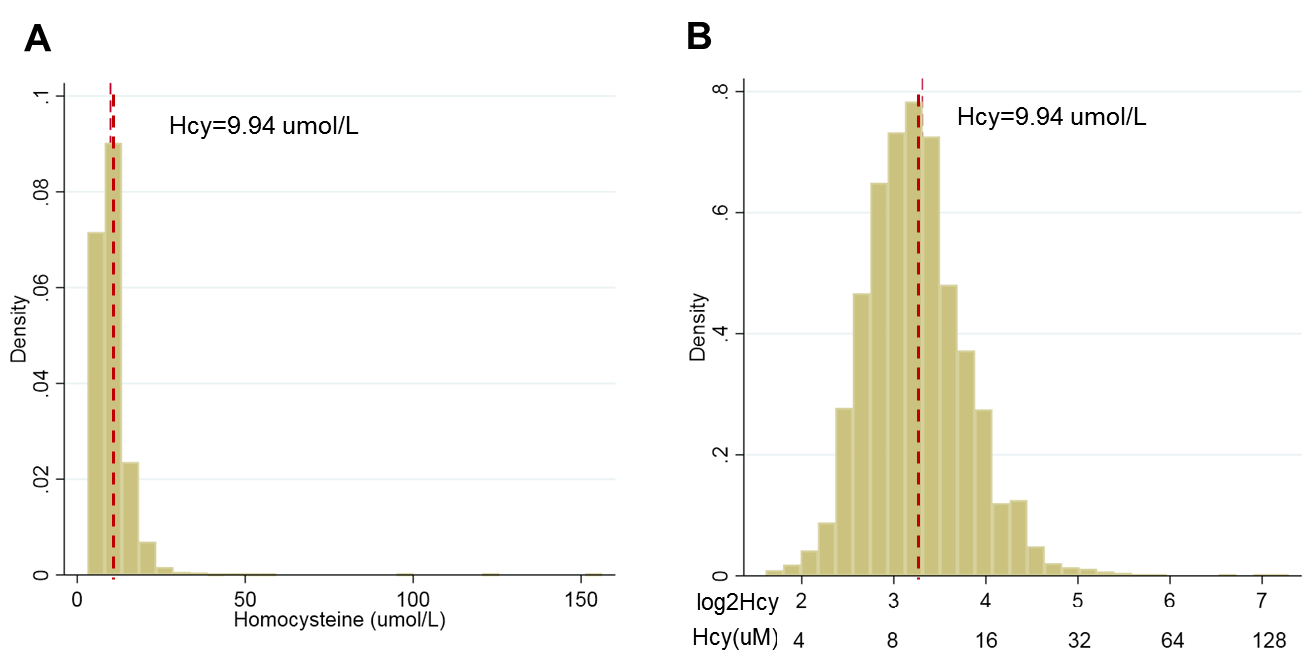


(A) Raw data was skewed distribution (B) log2 transformed Hcy was normal distribution. The weighted mean level of serum Hcy was 9.94 µmol/L (dashed line)

## Supplementary Figure 2. Box plots of serum Hcybysurveycycle (A) and (gender)


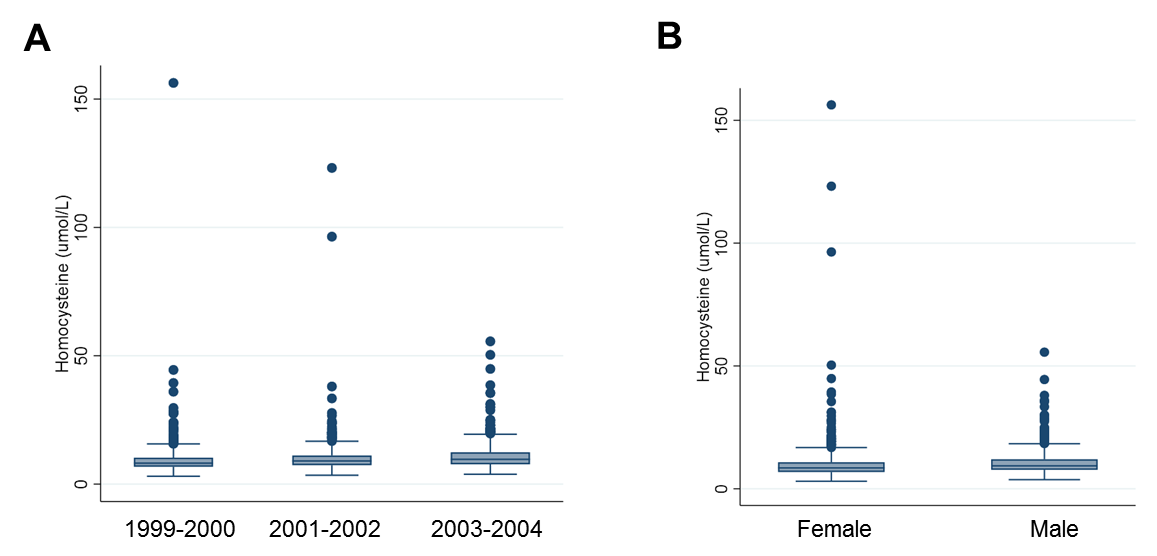


Y-axis represents the serum levels of Hcy (nmol/L) which were comparable across study years (A) and gender (B).
